# Effects of landscape, resource use, and body size on genetic structure in bee populations

**DOI:** 10.1002/ece3.11358

**Published:** 2024-05-12

**Authors:** Melissa Hernandez, Sevan Suni

**Affiliations:** ^1^ The University of San Francisco San Francisco California USA

**Keywords:** bee, body size, deforestation, dispersal, Euglossine, genetic structure, habitat

## Abstract

Quantifying genetic structure and levels of genetic variation are fundamentally important to predicting the ability of populations to persist in human‐altered landscapes and adapt to future environmental changes. Genetic structure reflects the dispersal of individuals over generations, which can be mediated by species‐level traits or environmental factors. Dispersal distances are commonly positively associated with body size and negatively associated with the amount of degraded habitat between sites, motivating the investigation of these potential drivers of dispersal concomitantly. We quantified genetic structure and genetic variability within populations of seven bee species from the genus *Euglossa* across fragmented landscapes. We genotyped bees at SNP loci and tested the following predictions: (1) deforested areas restrict gene flow; (2) larger species have lower genetic structure; (3) species with greater resource specialization have higher genetic structure; and (4) sites surrounded by more intact habitat have higher genetic diversity. Contrasting with previous work on bees, we found no associations between body size and genetic structure. Genetic structure was higher for species with greater resource specialization, and the amount of intact habitat between or surrounding sites was positively associated with parameters reflecting gene flow and genetic diversity. These results challenge the dominant paradigm that individuals of larger species disperse farther, and they suggest that landscape and resource requirements are important factors mediating dispersal.

## Graphical abstract

Quantifying genetic structure and levels of genetic variation are important to predicting the ability of populations to persist in human‐altered landscapes and adapt to future environmental changes. We assessed genetic diversity and genetic structure for seven Euglossine bee species that vary in resource specialization and body size. We found that genetic structure was higher for species with greater resource specialization, there was no association between body size and genetic structure, and the amount of intact habitat between or surrounding sites was positively associated with parameters reflecting gene flow and genetic diversity.

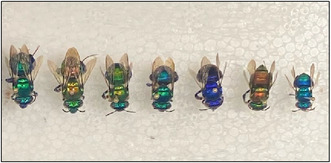

## 1 INTRODUCTION

As much as 75% of the global land surface has been modified by humans (Luyssaert et al., 2014). One of the most concerning forms of land modification is deforestation, which typically leads to fragmented landscapes that are characterized by small, isolated patches of forest surrounded by agriculture or human infrastructure. Deforestation is a leading cause of biodiversity loss worldwide, due to negative effects on abundance, species diversity, and genetic diversity (Schlaepfer et al., 2018).

Theory suggests that populations persisting in fragmented areas may experience genetic erosion before changes in abundance can be detected (Pflüger et al., 2019). Therefore, quantifying the genetic variability and genetic structure of populations living in fragmented areas is fundamental to understanding their ability to persist in human‐altered landscapes and adapt to future environmental changes. Genetic structure reflects a non‐random spatial distribution of genotypes, which occurs when gene flow is limited across space (Wright, 1943). Gene flow occurs via dispersal and maintains genetic diversity within populations (Franklin, 1980). Spatially limited gene flow often results in a pattern whereby populations become more genetically distinct as the distance between them increases, a pattern termed “isolation by distance” (Wright, 1943). Landscape features such as water bodies or mountains can also impede gene flow, a pattern called “isolation by resistance” (McRae, 2006). Populations that are isolated and for which dispersal is limited may be at higher risk of extinction due to loss of alleles via genetic drift, which lowers evolutionary potential (Frankel & Soulé, 1981).

Dispersal distances may be mediated both by individual characteristics and environmental effects (Baguette et al., 2012). Dispersal scales linearly with body size across many clades, including birds and mammals (Dawideit et al., 2009; Ottaviani et al., 2006), moths (Beck & Kitching, 2007), plants (Thomson et al., 2010), butterflies (Stevens et al., 2013), and bees (López‐Uribe et al., 2019). However, dispersal‐body size associations often show high variability within the groups assessed, and other species‐level characteristics may also be important such as life history traits (McCoy et al., 2010; Stevens et al., 2013), dispersal capacity (Hillman et al., 2014), diet breadth (Stevens et al., 2014), and other resource requirements (Bowler & Benton, 2005).

Environmental drivers of dispersal include resource availability (Baguette et al., 2012) and the extent of landscape connectivity among sites (Baguette et al., 2013). Larger organisms tend to have higher resource requirements than smaller organisms, so resource availability may more strongly influence dispersal propensity of larger organisms than smaller ones (Byers, 2000). In terms of landscape connectivity, physical barriers to movement and habitat quality throughout the landscape can both restrict dispersal (Manel & Holderegger, 2013). Negative effects of anthropogenically altered habitat on dispersal have been found for a range of species including small mammals (Ribeiro et al., 2021), birds (Björklund et al., 2010), bees (Jha & Kremen, 2013) and butterflies (Crawford et al., 2011; Takami et al., 2004). This may be due to higher mortality for animals that travel farther in between habitat fragments (Bonelli et al., 2013; Lucas et al., 1994; Mennechez et al., 2003). Other studies reveal little evidence of restricted dispersal across anthropogenically altered areas for organisms including bats (Richardson et al., 2021), plants (Culley et al., 2007), and other bee species (Suni, 2017). Urban areas may even act as a conduit for movement in some species (Ballare & Jha, 2021; Miles et al., 2019). Therefore, understanding interplay among body size, resource requirements, and landscape in mediating dispersal distances is critical given ongoing and projected anthropogenic landscape changes.

Bee pollinators may be particularly vulnerable to negative effects of habitat fragmentation due to their haplodiploid genetic systems, which render their effective population sizes no more than 75% that of equally sized diploid populations (Whiting & Whiting, 1925). Widespread population declines due to habitat loss have been reported for many bee species (LeBuhn & Vargas Luna, 2021; Potts et al., 2010), and these may occur via the loss of floral resources or nesting areas (Carvell et al., 2006; Cohen et al., 2021), greater energetic costs associated with travel (Andrieu et al., 2009), or heat stress (Aguirre‐Gutiérrez et al., 2017; Suni & Dela Cruz, 2021). Body size and resource specialization have been proposed as important traits that may mediate responses of bees to habitat loss. Larger bees are potentially able to cross larger degraded areas, but they also require larger areas of forage to persist (Harrison & Winfree, 2015). Meta‐analyses based on mark‐recapture and genetic data suggest larger bees travel farther (Greenleaf et al., 2007; López‐Uribe et al., 2019), but explicit tests of how body size and landscape may jointly influence dispersal in bees are lacking. Regarding resource specialization, current evidence on how generalist species may respond to habitat loss is contradictory. Generalists are predicted to be more resistant to negative effects of habitat loss due to their ability to use resources in more patches (Johnson et al., 2000). However, body size might affect how resistant populations are to habitat loss; small generalist bees have been found to be more affected by habitat loss than small specialists (Bommarco et al., 2010). In addition, diet specialization has often been used as the measure of niche breadth, but other resource requirements may also influence dispersal (Bowler & Benton, 2005). Taken together, this past research motivates the investigation of potential intersections of landscape and species‐level traits on parameters that mediate bee dispersal in fragmented landscapes.

Here, we examined drivers of genetic structure and genetic diversity for seven species of bees in the tribe Euglossini that vary widely in body size. Euglossine bees (also called Orchid Bees) are important pollinators of over 700 species of orchids and other tropical plants (Roubik & Hanson, 2004). Male Euglossine bees exhibit a unique behavior whereby they visit orchids and other plants to collect volatile compounds that are used in sexual chemical signaling when emitted during courtship behavior (Eltz et al., 2005). Euglossine bees have previously been found to show weak genetic structure over tens to hundreds of kilometers (Boff et al., 2014; da Rocha Filho et al., 2013; Soro et al., 2017; Suni, 2017; Suni et al., 2014; Suni & Brosi, 2012; Suni & Hernandez, 2023; Zimmermann et al., 2011). However, that previous work used microsatellite loci, which may provide less insight into patterns of genetic structure than a large number of SNP loci would (Gärke et al., 2012). To understand if landscape characteristics and species‐level traits are associated with genetic structure and diversity, we developed SNP loci for each of seven species in the genus *Euglossa* that vary in body size. We then tested the following predictions: (1) deforested areas restrict gene flow; (2) larger species have lower genetic structure; (3) species with greater resource specialization have higher genetic structure; and (4) sites surrounded by more intact habitat have higher genetic diversity.

## 2 MATERIALS AND METHODS

### 2.1 Field sampling

**FIGURE 1:**
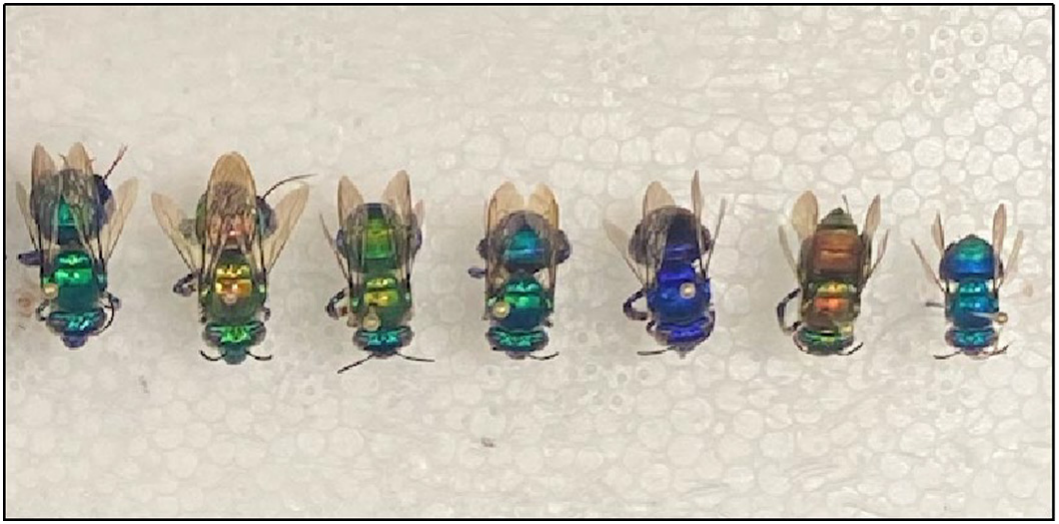
The seven Euglossine species sampled, along with their body sizes. From left: *Euglossa imperialis* (15 mm), *Euglossa flammea* (14 mm), *Euglossa championi* (13 mm), *Euglossa maculilabris* (12 mm), *Euglossa mixta* (11 mm), *Euglossa dodsoni* (10 mm), and *Euglossa sapphirina* (9 mm).

**FIGURE 2:**
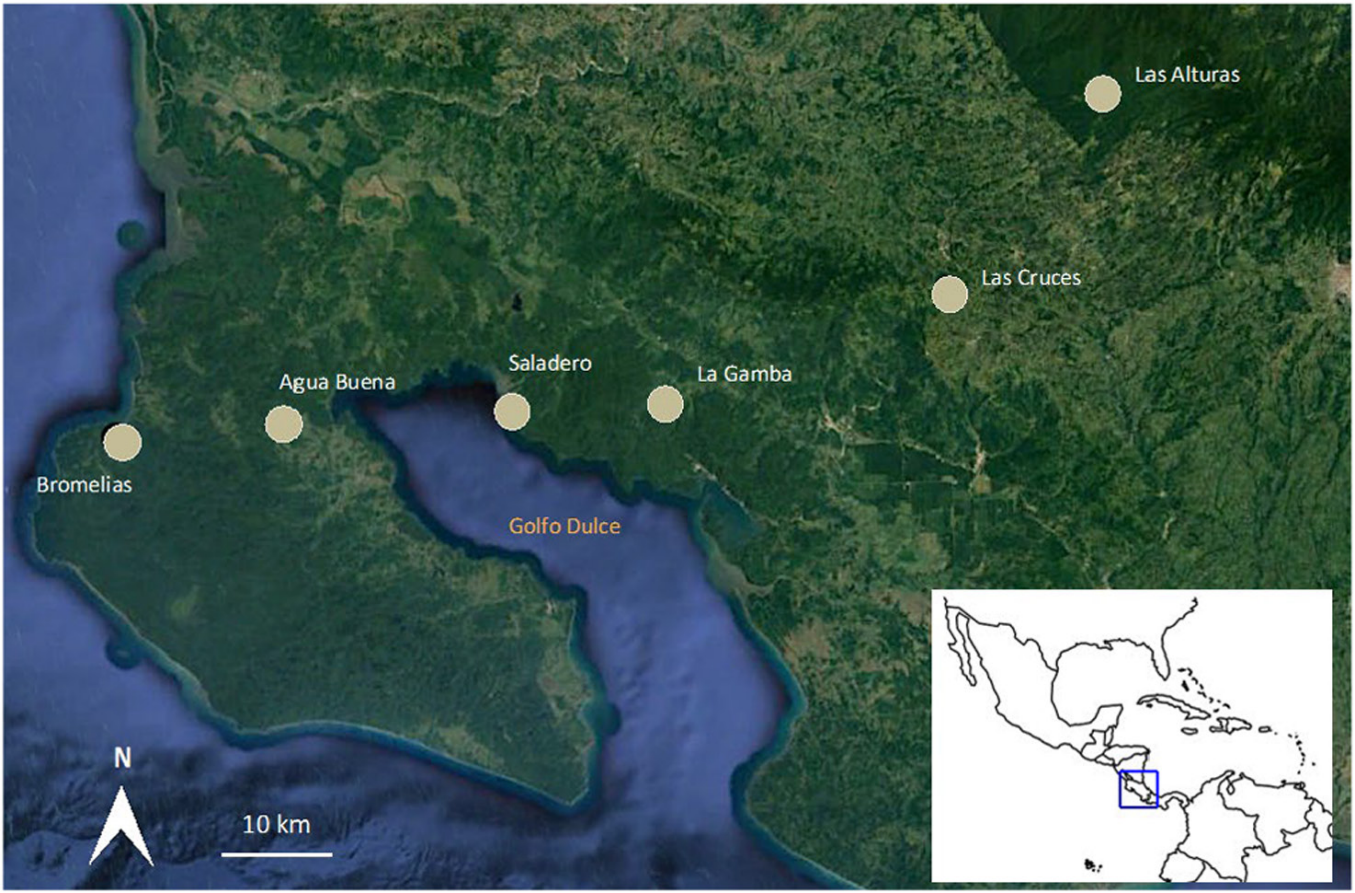
Study area in Southern Costa Rica, at which seven bee species in the genus *Euglossa* were obtained for an analysis of their genetic structure. Sites extend from costal sites on the Osa Peninsula (bottom left) to a forested site at 1420 m above sea level (top right). Image from Google Earth Pro v. 7.3.4.8248.

We sampled bees of seven species that range in body length from 9 to 15 mm (Figure 1) at six sites throughout southern Costa Rica in May and June of 2019 (Figure 2, Table 1). The sites and dates on which we sampled included the Las Alturas Biological Research Station (5/30/19), the Las Cruces Biological Research Station (5/18/19 and 5/20/19), the La Gamba Biological Research Station (6/3/19 and 6/4/19), the Saladero Ecolodge (6/5/19–6/7/19), the Bromelias Ecolodge (6/2/19), and a site at the northern part of the Osa Peninsula at which local landowners provided permission to sample (Agua Buena; 6/1/19; see Figure 2). The species sampled vary in their resource specialization, with the number of orchid morphospecies visited ranging from 6 to 20 (Roubik & Hanson, 2004; Table S1). The landscape in this area is comprised of forest fragments, pastureland, palm oil plantations, and small towns. Extensive deforestation occurred in the 1950s following European settlement and reduced forest cover to 25% by the 1990s, but pollen and charcoal analyses from lake‐sediment cores suggest continuous occupation and some forest clearing by indigenous people over a 3000‐year period (Clement & Horn, 2001).

**TABLE 1:** For each site at which bee species in the genus *Euglossa* were sampled in southern Costa Rica, the GPS coordinates, the mean annual temperature (MAT) in Celsius, the mean annual precipitation (MAP) in mm, percent of the landscape within a circle of radius 24 km that was forested (Tree), the species sampled (Species), the body size of the species in mm (Body size), the number of orchid morphospecies visited (Orchids), the number of specimens (N), the expected heterozygosity (*H*_e_), and percent of loci that were polymorphic (% Poly).

Site	Lat and Lon	MAT	MAP	Tree	Species	Body size	Orchids	*N*	*H*_e_	% poly
Agua Buena	8.694056 −83.521707	25.8	4108	67.4	*Eug. sapphirina*	9	6	8	0.15	0.12
Bromelias	8.685824 −83.662379	25.8	4460	44.7	*Eug. sapphirina*	9	6	14	0.15	0.14
La Gamba	8.702278 −83.203795	25.7	3959	61.1	*Eug. sapphirina*	9	6	20	0.13	0.14
Las Alturas	8.9453785 −82.833405	19.3	2997	76.0	*Eug. sapphirina*	9	6	8	0.15	0.11
Las Cruces	8.7875442 −82.964662	20.2	3283	64.3	*Eug. sapphirina*	9	6	4	0.13	0.08
Saladero	8.697707 −83.330522	25.9	4374	64.2	*Eug. sapphirina*	9	6	53	0.17	0.22
Agua Buena	8.694056 −83.521707	25.8	4108	67.4	*Eug. dodsoni*	10	14	4	0.18	0.08
Bromelias	8.685824 −83.662379	25.8	4460	44.7	*Eug. dodsoni*	10	14	5	0.18	0.09
La Gamba	8.702278 −83.203795	25.7	3959	61.1	*Eug. dodsoni*	10	14	25	0.20	0.16
Las Cruces	8.7875442 −82.964662	20.2	3283	64.3	*Eug. dodsoni*	10	14	7	0.21	0.13
Saladero	8.697707 −83.330522	25.9	4374	64.2	*Eug. dodsoni*	10	14	24	0.22	0.16
La Gamba	8.702278 −83.203795	25.7	3959	61.1	*Eug. mixta*	11	18	2	0.10	0.02
Las Alturas	8.9453785 −82.833405	19.3	2997	76.0	*Eug. mixta*	11	18	23	0.27	0.12
Las Cruces	8.7875442 −82.964662	20.2	3283	64.3	*Eug. mixta*	11	18	23	0.23	0.10
Saladero	8.697707 −83.330522	25.9	4374	64.2	*Eug. mixta*	11	18	2	0.17	0.04
Las Alturas	8.9453785 −82.833405	19.3	2997	76.0	*Eug. maculilabris*	12	9	32	0.28	0.09
Las Cruces	8.7875442 −82.964662	20.2	3283	64.3	*Eug. maculilabris*	12	9	5	0.25	0.05
Agua Buena	8.694056 −83.521707	25.8	4108	67.4	*Eug. championi*	13	11	6	0.15	0.12
Bromelias	8.685824 −83.662379	25.8	4460	44.7	*Eug. championi*	13	11	22	0.13	0.14
La Gamba	8.702278 −83.203795	25.7	3959	61.1	*Eug. championi*	13	11	25	0.15	0.21
Las Alturas	8.9453785 −82.833405	19.3	2997	76.0	*Eug. championi*	13	11	18	0.15	0.20
Las Cruces	8.7875442 −82.964662	20.2	3283	64.3	*Eug. championi*	13	11	26	0.14	0.22
Saladero	8.697707 −83.330522	25.9	4374	64.2	*Eug. championi*	13	11	24	0.15	0.23
La Gamba	8.702278 −83.203795	25.7	3959	61.1	*Eug. flammea*	14	8	4	0.28	0.06
Las Alturas	8.9453785 −82.833405	19.3	2997	76.0	*Eug. flammea*	14	8	8	0.31	0.07
Las Cruces	8.7875442 −82.964662	20.2	3283	64.3	*Eug. flammea*	14	8	10	0.28	0.07
Saladero	8.697707 −83.330522	25.9	4374	64.2	*Eug. flammea*	14	8	3	0.21	0.04
Agua Buena	8.694056 −83.521707	25.8	4108	67.4	*Eug. imperialis*	15	20	8	0.16	0.08
Bromelias	8.685824 −83.662379	25.8	4460	44.7	*Eug. imperialis*	15	20	26	0.17	0.14
La Gamba	8.702278 −83.203795	25.7	3959	61.1	*Eug. imperialis*	15	20	25	0.13	0.11
Las Alturas	8.9453785 −82.833405	19.3	2997	76.0	*Eug. imperialis*	15	20	2	0.09	0.03
Las Cruces	8.7875442 −82.964662	20.2	3283	64.3	*Eug. imperialis*	15	20	1	NA	NA
Saladero	8.697707 −83.330522	25.9	4374	64.2	*Eug. imperialis*	15	20	26	0.14	0.11

*Note*: Sampling dates include 5/20/2019 for Las Alturas, 5/31/2019 for Las Cruces, 6/1/2019 for Agua Buena, 6/2/19 for Bromelias, 6/3 & 6/4/2019 for La Gamba, and 6/6 and 6/7/2019 for Saladero. Temperature and precipitation data for each site were obtained from www.worldclim.org at a spatial resolution of 2.5 min.

To attract bees, we used the chemical baits 1,8‐cineole and methyl salicylate. These chemical baits mimic the natural fragrances emitted by orchids (Janzen, 1981). They have been reported to attract both generalist and specialist species, including the species used in this study (Roubik & Hanson, 2004). We saturated cotton balls with chemical baits and used thumb tacks to attach them to tree trunks approximately 1.5 m off the ground, between the hours of 9 am and 12 pm on sunny days, and in forest fragments between 0 and 93 m from forest edges. We netted bees as they arrived at baits, and we stopped sampling when no more bees arrived after 15 min. Bees were killed using the fumes of ethyl acetate in vials and then transferred to vials containing 100% ethanol on the same day. Samples were then transported back to the University of San Francisco for curation and DNA extraction. Bees were pinned and then identified by examining the velvet area, a patch of dense hair on the tibial tuft, as well as other species‐specific characteristics (Roubik & Hanson, 2004).

### 2.2 DNA sequencing and SNP calling

We extracted genomic DNA from one or two middle legs of each specimen (two legs for the smallest species) using DNeasy Blood and Tissue Extraction Kits (Qiagen). We quantified DNA concentration using a Qbit 2.0 fluorometer (Thermo‐Fisher) and then used 100 ng of DNA per individual to prepare ddRADseq libraries using a protocol modified from Poland et al. (2012), as follows. We digested DNA with the enzymes PstI and MspI (New England Biolabs) and then ligated unbarcoded adaptors that were synthesized by IDT (Integrated DNA Technologies) onto the sticky ends. We cleaned ligation products with Agencourt AMPure XP beads (Beckman Coulter), which we then used as templates for PCR. We performed PCR in 96‐well plates with each well containing one sample and one of 285 uniquely barcoded TruSeq primer pairs that had been synthesized by the University of California San Francisco Center for Advanced Technology (UCSF CAT). We used an AccuBlue DNA Concentration Kit (Biotium) to quantify DNA and then pooled 40 ng of each sample. We cleaned pooled DNA using Agencourt AMPure XP beads and then size‐selected the DNA (300–500 bp) using a Blue Pippin (Sage Science). We confirmed our success in obtaining accurate target fragment size distributions using a TapeStation 4200 (Agilent). We cleaned the pooled, size‐selected DNA using a Monarch PCR & DNA cleanup kit (NEB) and then performed 150‐bp paired‐end sequencing on a NovaSeq 6000 (Illumina) at the UCSF CAT. To maximize sequencing coverage, we performed two NovaSeq runs, such that all individuals of a given species were run on the same NovaSeq. The first run consisted of 284 samples belonging to *Eug. imperialis*, *Eug. championi*, and *Eug. dodsoni* (Table S2). The second run consisted of 285 samples belonging to *Eug. flammea*, *Eug. maculilabris*, *Eug. mixta*, and *Eug. sapphirina* (Table S2), and it also included additional Euglossine species of a different genus that were not included in this study.

We obtained demultiplexed sequences from the UCSF CAT. We assessed the quality of the sequencing run using the software FastQC v.0.11.8 (Andrews, 2010), and we compared forward (R1) and reverse (R2) raw fastq files for a subset of samples, checking for per base sequence quality, per‐sequence guanine‐cytosine (GC) content, and adapter content. Following the initial quality check, we used the software Stacks v. 2.54 (Catchen et al., 2011, 2013) to process the sequence data. First, we cleaned the raw Illumina reads using the *process_radtags* program. We applied filters that discarded reads for which the restriction enzyme cut‐site for MspI or PstI was not intact, reads with Illumina TruSeq adapter contamination, and reads with quality scores (Phred33) below 10 within a sliding window of 15% of the read length. We then used the *denovo_map.pl* pipeline to identify orthologous loci across individuals for each species separately. We performed STACKS parameter optimization for each species using a small subset of individuals, following (Paris et al., 2017). We chose the following parameter combination: *m* = 3, *M* = 2, *n* = 3 for each species, where *m* is the minimum stack depth parameter that controls the number of raw reads required to form an initial stack, *M* is the distance allowed between stacks, which represents the number of nucleotides that may be different between two stacks in order to merge them, and *n* is the distance allowed among catalog loci. We also set the following filtering options: ‐‐*paired* to assemble contigs from paired‐end reads and ‐‐*rm‐pcr‐duplicates* to retain a single set of paired‐end reads of the same insert length. We set *max‐obs‐het* to 0 as in Alonso‐Garcia et al. (2020), to process only nucleotide sites at loci in which the maximum observed heterozygosity was 0 and to remove paralogous loci. To minimize the number of retained loci that would be missing in some populations, we re‐ran the last step of the *denovo_map.pl* pipeline, the *populations* program, to retain only polymorphic loci present at certain frequencies. We enabled *‐‐min‐populations* so that a locus had to be present in at least two fewer the number of sampling sites, and we set *‐‐min‐samples‐per‐pop* to 0.75. We limited analyses to the first SNP per locus using *‐‐write‐single‐snp*, and we used the *‐‐fstats* option in the populations program to estimate expected heterozygosity, the number of private alleles, and the percent of loci that were polymorphic for each species within each site. As an additional measure of genetic diversity, we calculated allelic richness using the R package Hierfstat (Goudet, 2005).

### 2.3 Landscape characterization

To estimate the forest percent surrounding each sampling location and between locations, we used ArcGIS v.2.4 (Esri, Redlands, CA). We used the Esri 2020 Land Cover dataset that corresponded to scene 17P (Karra et al., 2021) to obtain forest cover of the study region. We quantified the amount of forest cover within a circle of radius 24 km for each sampling location (Figure S1). We chose this radius because Euglossine bees are capable of traveling over tens of kilometers in a single day (Janzen, 1971), and because this was the Euclidian geographic distance between the farthest edge of the Las Cruces site to where we sampled at Las Alturas. Those two sites are our longest‐term study sites between which we have been monitoring Euglossine bee genetic structure for over 12 years. To estimate the amount of forest between pairs of sampling locations we first used ArcGIS to calculate Euclidian (straight‐line) geographic distances between all possible site pairs. Euclidian distances are the shortest distance between sites and may traverse water. We also calculated “Broken‐stick” geographic distances as in Davis et al. (2010), which are the shortest overland distances between two sites. For both types of distances, we overlaid rectangles of width 1000 m and calculated the amount of forest between each pair of sites. We centered rectangles at each pair of sites and quantified the percent of the area that was forested within that rectangle (Figure S1). Many sites are located near the coastlines of the Golfo Dulce or the Pacific Ocean. We did not clip the circular or rectangular buffers to the coastline if they extended into the water, so water was included as deforested area. We did this to obtain a realistic estimate of the proportion of forest cover relative to other land cover types and to reflect possible Euglossine bee flight paths, since some Euglossine species seem to have restricted dispersal over large bodies of water (da Rocha Filho et al., 2013).

### 2.4 Population and landscape genetics

To determine if deforested areas restrict gene flow (prediction 1), we used Maximum Likelihood of Population Effects (MLPE) mixed models to determine the effects of landscape on genetic structure while taking the geographic distance between pairs of sites into account. MLPE models are emerging as a powerful analytical approach in landscape genetics that permits theoretic model selection (Jha & Kremen, 2013; Row et al., 2017). The MPLE approach uses pairwise individual‐based genetic distances as a response variable, landscape resistances, and geographic distance as fixed effects, and includes a random effect matrix of pairwise individual comparisons that accounts for the non‐independent nature of the pairwise dataset (Clarke et al., 2002). Our models included genetic distance between pairs of individuals as the dependent variable, the amount of forest and geographic distance between sites as independent variables, and the individuals compared as a random effect.

We used Hamming distance as our measure of genetic distance between individuals. Hamming distance measures the dissimilarity between two strings of equal length (Hamming, 1950). It has long been used in information theory and it is becoming more widely used in population genetics (Wang et al., 2015). Hamming distance is especially useful when studying haploid organisms (Widhelm et al., 2021), such as the male bees we used in this study. We calculated the Hamming distance among all pairs of individuals separately for each species. First, we used Stacks to output a genepop file containing SNP genotypes, which we then converted into a genind object using the Adegenet package in R (Jombart, 2008). Then, we used a series of custom scripts that leveraged the R packages Hierfstat, tseries (Trapletti & Hornik, 2022), ResistanceGA (Peterman, 2018), and nlme (Pinheiro et al., 2017) to calculate genetic distance and implement the MLPE models (see “Data Availability Statement,” below for how to access custom scripts).

To implement the MLPE approach, we ran a set of seven generalized least square (GLS) models for each species separately. Code that uses generalized least squares (GLS) models to implement the MLPE covariance structure is available at: https://github.com/nspope/corMLPE. We ranked models according to their Akaike Information Criteria corrected for sample size (AIC_c_), as in Balbi et al. (2018). We report estimates and *p*‐values for fixed effects for models for which the difference from the model with the greatest negative log likelihood was <2. Our models were as follows: a full model that included Euclidian geographic and forest distances as the independent variables, a model that included only Euclidian geographic distance, a model that included only forest geographic distance, a full model that included broken‐stick geographic and forest distances as the independent variables, a model that included only broken‐stick geographic distance, a model that included only broken‐stick forest distance, and an intercept only model. To understand if male Euglossine bees of some species disperse away from their natal areas, but do not travel across the whole geographic areas sampled, we also ran a second set of models for each species using datasets that included comparisons only between samples from different sites (no within‐site comparisons). We then evaluated if the relationship between genetic and geographic distance differed between these two sets of models. We ran MLPE models for species from which at least three individuals had been sampled from at least four sites (Table 1).

To determine if body size or resource generalization predicts genetic structure (predictions 2 & 3), we first calculated the average genetic distance between pairs of individuals for each pair of sites, for each species. We then used this as the dependent variable in linear mixed models implemented using the lme4 package in R (Bates et al., 2015). We ran two models, one with body size as the independent variable, and one with resource specialization as the dependent variable, and we included the pair of sites between which average genetic distance was calculated as the random effect. To assess resource specialization, we compiled the number of morphospecies and genera of orchids visited for each species from records reported in Roubik and Hanson (2004). We tested for statistical significance of the independent variable of each model using likelihood ratio tests on nested models. In the results section we report estimates from the best model chosen via backward model selection, and chi‐square and associated *p*‐values from likelihood ratio tests. Table S3 shows the dataset used in this analysis. We tested for an association between body size and resource specialization using the cor.test function in R.

To determine if sites that were surrounded by more forest had higher genetic diversity (prediction 4), we ran linear mixed models implemented using the *lme4* package in R (Bates et al., 2015; R Core Team, 2019). Either expected heterozygosity, the number of private alleles, or allelic richness was the dependent variable. We modeled those dependent variables as a function of the forest percent surrounding sites at a radius of 24 km, and we included sample size as a covariate and species as a random effect. We used a dataset that included only species‐site combinations that had at least four individuals sampled for this analysis, and tested for significance of the independent variables using likelihood ratio tests on nested models.

## 3 RESULTS

The first sequencing run produced 467,504,244 reads (mean per sample = 1,663,716) and the second run produced 679,177,300 reads (mean per sample = 2,451,904). After initial quality filtering, we retained 207,471,708 reads in the first run (mean per sample = 738,333) and 508,060,286 reads in the second run (mean per sample = 1,834,153). After genotyping and quality control, our final sample included 493 bees that represented an average of 15 bees per species per site (Table 1). The de novo assembly generated a mean of 82,670 ± 35,080 loci across the Euglossine bee species (Table S2). Of these, the mean number of polymorphic loci was 6998 ± 4124, which represented a mean of 73,656 ± 62,300 SNPs per species. After the filtering to require that loci were present in several populations (see Section 2), the mean number of assembled loci was 8640 ± 7329, and the mean number of polymorphic loci was 2994 ± 2477 (Table S2).

The average genetic distance among individuals between pairs of sites ranged from 0.0017–0.18 for all species, and the average for each species across all site pairs ranged from 0.034 to 0.1. We found support for prediction (1), that deforested areas restrict gene flow. For all species, there was a significant negative relationship between the amount of forest between pairs of sites and the genetic distance among individuals, when pairwise comparisons among bees within sites were included in MLPE models (Table S4). There was variation across species in whether they exhibited isolation by distance. There was a significant positive relationship between genetic and geographic distance for species with the lowest resource specialization (*Eug. sapphirina* and *Eug. flammea*) but not for the more generalized species (*Eug. dodsoni*, *Eug. championi*, and *Eug. imperialis*; Table S4). MLPE models that omitted pairwise comparisons among bees within sites revealed a pattern of isolation by distance for all species (Table S5).

**FIGURE 3:**
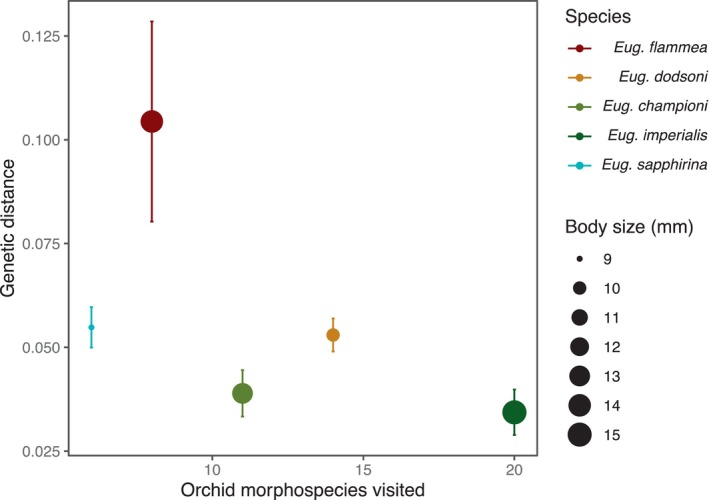
For each species, genetic distance averaged across individuals within sites and then averaged across sites is plotted against the number of orchid morphospecies visited by that species (as reported by Roubik & Hanson, 2004). Error bars represent standard errors calculated from within site averages. Colors represent different species and the size of the points reflects differences in body size.

We found no support for prediction (2), that body size predicts genetic structure. The genetic distance among pairs of individuals was not statistically associated with body size (*χ* ^2^ = 0.77, *p* = .78, Table S3). However, we found support for prediction (3), that resource specialization predicts genetic structure. The number of orchid morphospecies visited was negatively related to the average genetic distance among individuals within species (Est. = −0.002, *χ* ^2^ = 5.0, *p* = .025; Table S3; Figure 3). There was no correlation between resource specialization and body size (*t* = 0.8, *p* = .46).

**FIGURE 4:**
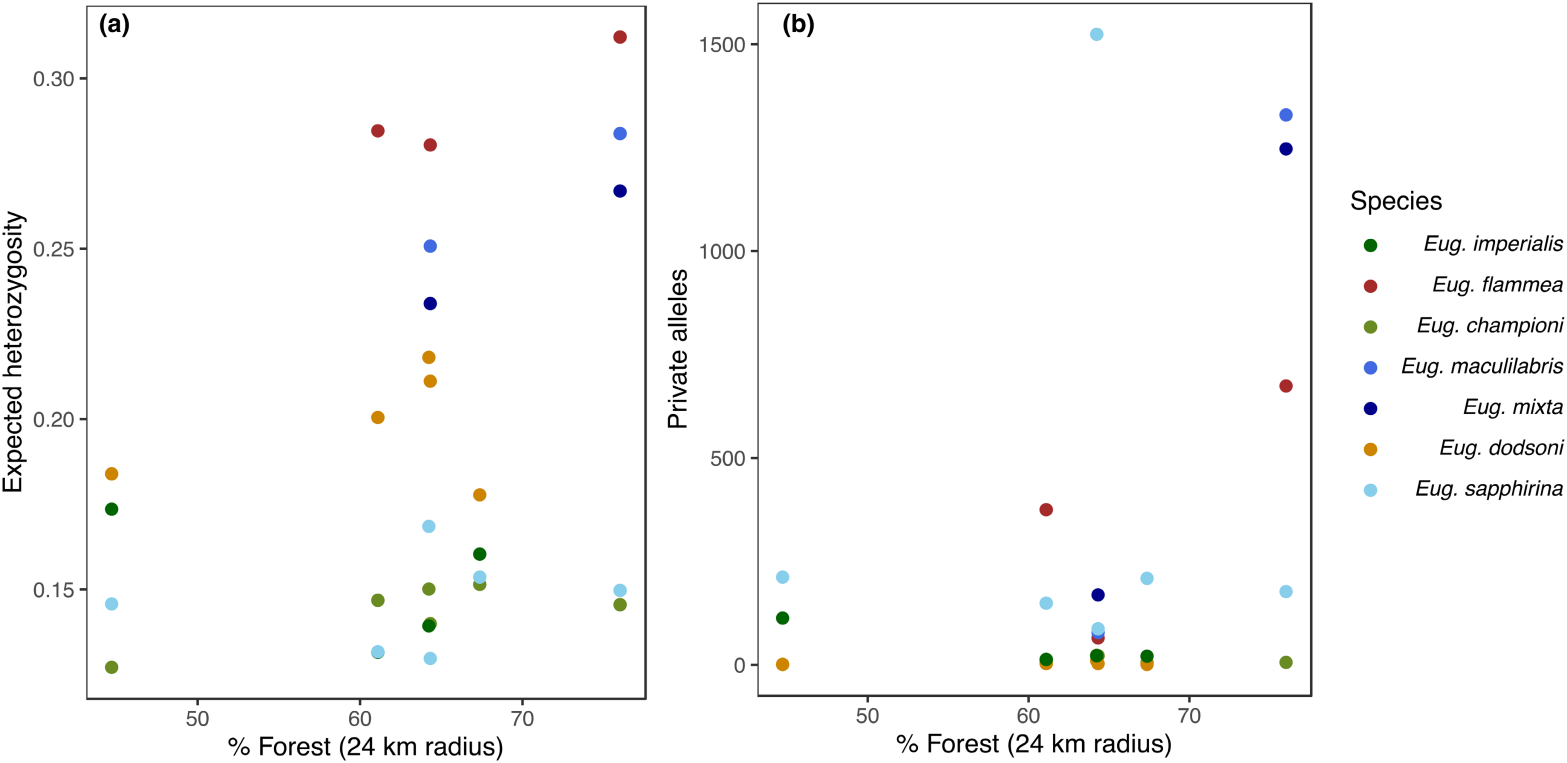
For each species, expected heterozygosity within sites (panel a) or the number of private alleles (panel b) is plotted against the percent of forest surrounding sites at a radius of 24 km from the sampling location. Colors represent different species of Euglossine bees (genus *Euglossa*) sampled from six sites in southern Costa Rica.

Euglossine bee species varied in their genetic diversity (Table 1; Figure 4). Across species and sites, means (±SD) were as follows: 0.19 ± 0.057 for expected heterozygosity, 234 ± 427 for private alleles, and 1.4 ± 0.18 for allelic richness. We found support for prediction (4), that the amount of intact habitat around sites positively affected genetic diversity. There was a trend towards increased expected heterozygosity in sites surrounded by more forest (*χ* ^2^ = 3.0, *p* = .084, Table 1, Figure 4a), although this trend was not significant. Sites that were surrounded by more forest had more private alleles (Est. = 12.9, *χ* ^2^ = 4.44, *p* = .035; Table 1, Figure 4b). Allelic richness did not vary with the amount of forest surrounding sites (*χ* ^2^ = 1.9, *p* = .17, Table 1).

## 4 DISCUSSION

Our joint analysis of individual traits and landscape effects on dispersal reveals patterns that contradict the dominant paradigm found for bees regarding body size and highlights the potential importance of resource specialization in influencing dispersal in fragmented landscapes. We present a systematic investigation of morphological and landscape drivers of genetic structure for seven bee species within a clade, as well as an assessment of how genetic diversity varies with the amount of intact habitat surrounding sites. We found evidence that forested landscape facilitates gene flow, as genetic distances among pairs of bees were higher between sites separated by less forest. We also found that genetic structure was not related to body size, but that it was related to resource specialization. Bee species that were more specialized in the orchid morphospecies from which they collected floral fragrances had higher genetic structure. Finally, we found evidence that the amount of forested area surrounding sites was positively associated with the genetic variability of bees in those sites.

The movement of animals can be altered in landscapes that have been fragmented (Fahrig, 2007). This includes the movement of flying organisms that may not be impeded by physical barriers but that may still experience risks associated with travel over degraded or open areas (Caizergues et al., 2003; Vidal & Rendón‐Salinas, 2014). For Euglossine bees, dispersal over deforested areas may be influenced by the extent to which they are heat‐tolerant (Roubik, 1993), as deforested areas may be much hotter than intact forest (Mantyka‐pringle et al., 2012). Deforested or open areas may also pose greater predation risks if it compromises the ability to camouflage (Coker et al., 2009). Past work has revealed restricted dispersal across water for some bee species in the genus *Euglossa* (Boff et al., 2014; da Rocha Filho et al., 2013). Therefore, it is not surprising that distances that traced water bodies better explained genetic structure for most species, especially for the species with the highest gene flow across the landscape.

The positive association we found between genetic and geographic distance is somewhat consistent with past work on Euglossine bees. Mark‐recapture observations of bees in the genus *Euglossa* have documented high recapture rates over monthly time periods (Eltz et al., 1999; López‐Uribe et al., 2008). Other mark‐recapture efforts across sites have documented male bees traveling tens of kilometers within a period of days through intact forest (Pokorny et al., 2015). In addition, past population genetic studies have typically found evidence of restricted dispersal for species in *Euglossa* only for island populations (Boff et al., 2014; da Rocha Filho et al., 2013). For populations separated by land, mitochondrial COI genotyping found identical haplotypes on both sides of the Andes mountains (Dick et al., 2004). Microsatellite genotyping found a low genetic structure for *Eug. dilemma* across 130 km (Zimmermann et al., 2011), *Eug. dilemma* and *Eug. viridissima* across 114 km (Soro et al., 2017), *Eug. imperialis* across 226 km (Suni, 2017), and *Eug. championi* across 14 km (Suni & Brosi, 2012) and across 80 km (Suni et al., 2014). Our work differs from past work in that it leverages hundreds to thousands of SNP loci per species to assess genetic structure. The use of more powerful markers may explain our ability to detect significant isolation by distance and a signal that forest promotes dispersal. This discrepancy between microsatellite and SNP‐based results is consistent with past work that found higher sensitivity of SNPs for detection of genetic structure using the same DNA (Zimmerman et al., 2020).

The lack of an association between body size and genetic structure contrasts with what has been found previously for bees at a taxonomically wide scale. A significant positive relationship was found between body size and homing or foraging distance for 62 bee species from six families (Greenleaf et al., 2007). That study compiled observational data of short‐term movement patterns and did not include estimates of realized dispersal. A meta‐analysis that examined associations between body size, and estimates of genetic structure based on microsatellites, found high variation but an overall negative relationship between body size and genetic differentiation across 42 species of bees (López‐Uribe et al., 2019). At taxonomically narrower scales, lack of an association between body size and genetic structure has been observed. For example, genetic structure did not increase as body size decreased across four *Bombus* species (Knight et al., 2005), and body size was not a predictor of the strength of isolation by distance in stingless bees (Jaffé et al., 2016). This suggests that traits other than body size are also likely important drivers of genetic structure. Indeed, López‐Uribe et al. (2019) found that social species had lower genetic structure than solitary species, which could be due to higher levels of kin competition for social species when compared to solitary species (West et al., 2002). In our case, reports of nest sharing have been reported for species within the genus *Euglossa* (Augusto & Garófalo, 2004), so we posit that the avoidance of kin competition may not be a strong driver of genetic structure.

Our data suggest that species that are more generalized in their resource use either disperse farther or travel farther when foraging. This is consistent with some other work showing that resource specialization is associated with lower gene flow. For example, species that are more generalized in their resource requirements are expected to be able to disperse farther due to their ability to refuel *en route* (Bowler & Benton, 2005). However, an empirical survey of 740 species of varying tropic levels found no association between resource specialization and dispersal (Stevens et al., 2014). In addition, work specifically on bees also found no evidence that genetic structure is associated with the degree of diet specialization across 42 species (López‐Uribe et al., 2019). Though diet specialization is commonly used as a measure of niche breadth, resource requirements other than dietary requirements may also be important drivers of dispersal (Bowler & Benton, 2005). Our examination of the extent of floral generalization for fragrance collection revealed a positive association between the number of orchid morphospecies visited and gene flow. Many tropical plants are locally rare (Wills et al., 2006), and it is possible that species that are more generalized in the orchids they visit travel farther distances to acquire diverse bouquets of fragrances.

It may be important to consider the relationship between resource specialization and body size when considering the effects of each on genetic structure. If traits are correlated at the species level, determining how each contributes to movement may not be straightforward. Results from past work on associations between specialization and body size in bees are contradictory, with some studies finding that smaller species tend to be more specialized (Leonhardt & Blüthgen, 2012; Smith et al., 2019), and others finding that smaller species tend to be more generalized (Raiol et al., 2021). In our case, there was no association between body size and specialization, and we conclude that resource specialization is the main driver of genetic structure among the variables examined here.

It is worth noting that bees vary in their nesting behavior, with some species building aerial nests and others using pre‐existing cavities. Work on non‐Euglossine bees suggests that intact habitat may be particularly important for cavity nesters (Lima et al., 2020; Neame et al., 2013). However, some species of cavity nesters such as carpenter bees in the genus *Xylocopa* seem to be able to thrive in urban areas where human‐made cavities are present (Cane et al., 2006). For Euglossine bees, past work suggested that the costs of habitat destruction may be low for aerial nesters in previously deforested areas, if subsequent reforestation occurs. Abundances of Euglossine bees in Brazil were found to be high in secondary forest, which was attributed to there being more resin for nest construction (Becker et al., 1991). Regarding the species used in this study, there is variation in their nesting behavior (Table S1), and no apparent associations between nesting behavior and genetic structure. For example, there is variation in the nesting behavior among species that show lower genetic structure. *Euglossa dodsoni* and *Eug. championi* construct aerial nests (Eberhard, 1988; Riveros et al., 2009), while *Eug. imperialis* constructs nests in cavities that may be in the ground (Roberts & Dodson, 1967). This suggests nesting behavior may not be a strong driver of genetic structure for the bees examined here, but additional work on intersections between nesting behavior and deforestation on bee movement would be useful to strengthen any conclusions that can be drawn.

There was evidence that sites that were surrounded by less forest had lower genetic diversity. The susceptibility of populations to negative effects of habitat fragmentation depends on species‐specific characteristics, such as habitat specialization and dispersal capacity (Sekar, 2012; Slade et al., 2013), as well as habitat availability in the surrounding area (Peakall & Lindenmayer, 2006). Species with high dispersal capacity may be less likely to suffer from negative effects of fragmentation if they can utilize other habitat patches. This should result in the maintenance of gene flow among patches and genetic diversity within patches. Lower dispersal capacity but a network of accessible patches should result in a pattern of isolation by distance, as we found in this study. Low dispersal capacity and isolated fragments should lead to high genetic drift within patches and the loss of genetic diversity (Louy et al., 2007). With limited dispersal among fragments, genetic drift may quickly cause the loss of rare alleles in small populations (Allendorf, 1986). Our finding of significantly more private alleles in sites with more forest suggests that drift may be lower and effective population sizes higher in fragments surrounded by greater amounts of habitat. This supports other work that has documented decreases in genetic diversity with habitat loss across diverse taxa including mammals (Lino et al., 2019), plants (González et al., 2020), amphibians (Dixo et al., 2009), and insects (Bickel et al., 2006).

To our knowledge, this work is the first SNP‐based assessment of genetic structure in Euglossine bees, and our results highlight risks to populations associated with habitat fragmentation. In particular, genetic diversity was lower in areas with less intact forest, suggesting that these bee species may be at risk of further genetic erosion as habitat fragmentation continues. Indeed, a study that monitored genetic diversity over time for a species used in the current study, *Eug. championi*, found striking declines in genetic diversity over an 11‐year period (Suni & Hernandez, 2023). Our findings reveal new patterns than those found previously for Euglossine bees, which employed mitochondrial haplotypes or microsatellite loci to characterize genetic structure (Boff et al., 2014; da Rocha Filho et al., 2013; Dick et al., 2004; Soro et al., 2017; Suni, 2017; Suni et al., 2014; Suni & Brosi, 2012; Suni & Hernandez, 2023; Zimmermann et al., 2011). This is consistent with what has been found for bumble bees in temperate areas, where investigations of dispersal distances found discrepancies between patterns emerging from microsatellite versus SNP data (Lozier, 2014; Lozier et al., 2016). The inconsistency found across studies employing different markers therefore motivates investigation into additional population genetic studies in Euglossine bees and investigations into the extent to which ecological specialization mediates dispersal in bees more generally.

## AUTHOR CONTRIBUTIONS

**Melissa Hernandez:** Conceptualization (supporting); formal analysis (equal); investigation (equal); methodology (supporting); validation (equal); visualization (equal); writing – original draft (supporting); writing – review and editing (supporting). **Sevan Suni:** Conceptualization (lead); formal analysis (equal); investigation (equal); methodology (lead); resources (lead); software (lead); supervision (lead); validation (equal); visualization (equal); writing – original draft (lead); writing – review and editing (lead).

### OPEN RESEARCH BADGES

This article has earned an Open Data badge for making publicly available the digitally‐shareable data necessary to reproduce the reported results. The data is available at https://zenodo.org/records/10345245.

## BENEFIT‐SHARING STATEMENT

*Benefits generated*: Permission of local landowners was obtained prior to sampling. Results of scientific enterprises are being shared with landowners, including biological research stations and ecolodges that promote scientific research and engage with local communities. The contributions of local individuals to research are described in Section 2 and Acknowledgements.

## Supporting information


Data S1

## Data Availability

*Genetic data*: Datasets and code used to produce statistical results and figures as well as individual genotype data are available at: https://zenodo.org/records/10345245. Individual raw sequence reads are deposited in the SRA (BioProject ID: PRJNA880925). *Sample metadata*: Sample metadata, including georeferences in decimal degrees and dates of sampling events, are in Table 1.
