# Supplementary material for: Effects of landscape, resource use, and body size on genetic structure in bee populations
Source: Ecol Evol. 2024 May 12;14(5):e11358. doi: 10.1002/ece3.11358 (PMC11089087; doi:10.1002/ece3.11358)
Supplement: Supplementary file 1 — Data S1 [file ECE3-14-e11358-s001.pdf]

MOLECULAR ECOLOGY

Supporting Information for Online Publication for:

Effects of landscape, resource use, and body size on genetic structure in bee populations

Melissa Hernandez & Sevan Suni

Table of Contents:

**Table S1**	Page 2
**Table S2**	Page 3
**Table S3**	Page 4
**Table S4**	Page 7
**Table S5**	Page 9
**Figure S1**	Page 11

MOLECULAR ECOLOGY

Species	Body size	Orchids	Orchid species	Nesting
Eug. sapphirina	9	6	*Houlletia odoratissima, Mormodes, Notylia barkeri, Sievekingia fimbriata, Stanhope ecornuta, Trichocentrum capistratum*	Wood cavity
Eug. dodsoni	10	14	*Catasetum bicolor, Coeliopis hyacinthosma, Cycnoches guttulatum, Dressleria, Gongora horichiana, G. maculata, G. quinquenervis, Kefersteinia lacteal, Kegeliella, Mormodes igneum, Notylia linearis, Notylia sp, Peristeria, Sievekingia suavis*	Hard, nut shaped, on twig or branch
Eug. mixta	11	18	*Catasetum bicolor, C. thompsonii, Coryanthes speciosa, C. trifoliata, Cycnoches, Dichaea panamensis, Gongora quinquenervis, Kefersteinia costaricensis, Kegeliella, Mormodes atropurpureum, M. cartonii, M. igneum, M. colossus, M. maculatum, M. powellii, Notylia, Peristeria pendula, Sievekingia fimbriata*	Hollow stem or branch
Eug. maculilabris	12	9	*Coryanthes, Cycnoches, Dichaea, Kefersteinia, Lacaena spectabilis, Lycaste, Mormodes, Notylia, Peristeria*	Nest unknown
Eug. championi	13	10	*Cycnoches, Dichaea, Dressleria dilecta, D. eburnean, D. kerryae, Mormodes atropupureun, Noylia, Peristeria, Sobralia, Stanhopea cirrhata*	Dome under a leaf or in epiphyte
Eug. flammea	14	8	*Catasetum maculatum, Cycnoches egertonianum, Gongora, Peristeria leucoxantha, Sievekingia fimbriata, Stanhopea cirrhata, S. oculate, S. panamensis*	Ground cavity
Eug. imperialis	15	20	*Catasetum macrocarpum, C. saccatum, Coryanthes trifoliata, Cycnoches egertonianum, Dichaea, Gongora maculate, G. quinquenervis, Kefersteinia, Kegeliella kupperi, Mormodes, Notylia buchtienii, Peristeria, Polycycnis muscifera, Sobralia, Stanhopea candida, S. cirrhata, S. ecornuta, S. costaricensis, Trichocentrum maculatum, Trichopilia maculata*	Ground or rock cavity

Table S1. For each species, the body size, number and names of the orchid morphospecies visited, and the nesting habitat, as reported in Roubik & Hanson (2004).

MOLECULAR ECOLOGY

**Species**	**N**	**Mean depth of coverage**	**Retained reads**	**Assembled loci (pre-filtering)**	**Polymorphic loci (pre-filtering)**	**SNPs (pre-filtering)**	**Filtering**	**Assembled loci (post-filtering)**	**Polymorphic loci (post-filtering)**
E. sapphirina	107	47.27x	296,581,295	153,924	14,229	193,697	*p*=4, *r*=0.75	10,485	7,025
E. dodsoni	65	11.26x	32,220,986	51,257	948	9,434	*p*=3, *r*=0.75	292	124
E. mixta	51	19.55x	99,403,380	92,712	8,927	82,742	*p*=3, *r*=0.75	13,138	4,296
E. maculilabris	37	14.72x	64,371,382	85,557	7,532	26,620	*p*=1, *r*=0.75	19,355	4,448
E. championi	121	10.3x	92,578,500	66,226	5,326	88,882	*p*=4, *r*=0.75	2,238	1,298
E. flammea	25	18.35x	36,453,361	52,669	4,525	28,132	*p*=2, *r*=0.75	13,423	3,141
E. imperialis	88	10.3x	81,510,644	76,344	7,496	86,086	*p*=4, *r*=0.75	1,546	626

Table S2. Summary of Stacks output generated using the *process_radtags* and *denovo_map.pl* pipelines. Stacks was run for each orchid bee species separately. For each species, the sample size (N), the mean depth of coverage, number of reads retained after cleaning the raw genomic data using *process_radtags*, and the output from *denovo_map.pl*, including the number of assembled loci, polymorphic loci, and SNPs prior to filtering, the chosen parameter values, and the number of assembled loci and polymorphic loci post filtering. Polymorphic loci were filtered using the populations program and loci were processed if they were present in at least two fewer than the number of sampled sites (*p*) and at least in 75% of individuals (*r*). Species are listed from smallest to largest body size. Post filtering, there was one SNP per polymorphic locus. The *populations* program, which is embedded in the *denovo_map.pl* pipeline, used the parameter values m-3, M=1, n=2, where m is the minimum stack depth parameter that controls the number of raw reads required to form an initial stack, M is the distance allowed between stacks, which represents the number of nucleotides that may be different between two stacks in order to merge them, and n is the distance allowed among catalog loci.

MOLECULAR ECOLOGY

Species	Size (mm)	Orchids	Site 1	Site 2	For (E)	For (BS)	Km (E)	Km (BS)	GD
Eug. sapphirina	9	6	Agua Buena	Agua Buena	1	1	0	0	0.083
Eug. sapphirina	9	6	Agua Buena	Las Alturas	69.53	72.57	80.7	81.9	0.076
Eug. sapphirina	9	6	Agua Buena	Saladero	23.06	92.42	21	33.9	0.075
Eug. sapphirina	9	6	Bromelias	Agua Buena	94	94	15.4	15.4	0.054
Eug. sapphirina	9	6	Bromelias	Bromelias	1	1	0	0	0.049
Eug. sapphirina	9	6	Bromelias	Las Alturas	74.75	74.75	95.5	95.5	0.051
Eug. sapphirina	9	6	Bromelias	Las Cruces	71.83	89.23	77.5	78.1	0.054
Eug. sapphirina	9	6	Bromelias	Saladero	53.96	92.89	36.1	47.6	0.050
Eug. sapphirina	9	6	La Gamba	Agua Buena	53.6	94.95	34.9	39.5	0.027
Eug. sapphirina	9	6	La Gamba	Bromelias	66.65	94.54	50.4	53.2	0.026
Eug. sapphirina	9	6	La Gamba	La Gamba	1	1	0	0	0.023
Eug. sapphirina	9	6	La Gamba	Las Alturas	69.5	69.5	48.5	48.5	0.027
Eug. sapphirina	9	6	La Gamba	Las Cruces	81.66	81.66	27.7	27.7	0.028
Eug. sapphirina	9	6	La Gamba	Saladero	99.81	99.81	13.8	13.8	0.026
Eug. sapphirina	9	6	Las Alturas	Las Alturas	1	1	0	0	0.072
Eug. sapphirina	9	6	Las Alturas	Saladero	76.01	76.01	61	61	0.070
Eug. sapphirina	9	6	Las Cruces	Agua Buena	62.05	88.2	62	64.4	0.083
Eug. sapphirina	9	6	Las Cruces	Las Alturas	62.09	62.09	22.5	22.5	0.077
Eug. sapphirina	9	6	Las Cruces	Las Cruces	1	1	0	0	0.084
Eug. sapphirina	9	6	Las Cruces	Saladero	90.31	90.31	41.7	41.7	0.076
Eug. sapphirina	9	6	Saladero	La Gamba	99.81	99.81	13.8	13.8	0.024
Eug. sapphirina	9	6	Saladero	Saladero	1	1	0	0	0.069
Eug. dodsoni	10	14	Agua Buena	Agua Buena	1	1	0	0	0.069
Eug. dodsoni	10	14	Agua Buena	Saladero	23.06	92.42	21	33.9	0.048
Eug. dodsoni	10	14	Bromelias	Agua Buena	94	94	15.4	15.4	0.045
Eug. dodsoni	10	14	Bromelias	Bromelias	1	1	0	0	0.062
Eug. dodsoni	10	14	Bromelias	Las Cruces	71.83	89.23	77.5	78.1	0.064
Eug. dodsoni	10	14	La Gamba	Agua Buena	53.6	94.95	34.9	39.5	0.032
Eug. dodsoni	10	14	La Gamba	Bromelias	66.65	94.54	50.4	53.2	0.035
Eug. dodsoni	10	14	La Gamba	La Gamba	1	1	0	0	0.045
Eug. dodsoni	10	14	La Gamba	Las Cruces	81.66	81.66	27.7	27.7	0.044
Eug. dodsoni	10	14	Las Cruces	Agua Buena	62.05	88.2	62	64.4	0.061
Eug. dodsoni	10	14	Las Cruces	Las Cruces	1	1	0	0	0.097
Eug. dodsoni	10	14	Saladero	Agua Buena	23.06	92.42	21	33.9	0.045
Eug. dodsoni	10	14	Saladero	Bromelias	53.96	92.89	36.1	47.6	0.045
Eug. dodsoni	10	14	Saladero	La Gamba	99.81	99.81	13.8	13.8	0.039

Eug. dodsoni	10	14	Saladero	Las Cruces	90.31	90.31	41.7	41.7	0.060
Eug. dodsoni	10	14	Saladero	Saladero	1	1	0	0	0.056
Eug. championi	13	11	Agua Buena	Agua Buena	1	1	0	0	0.074
Eug. championi	13	11	Agua Buena	Las Alturas	69.53	72.57	80.7	81.9	0.059
Eug. championi	13	11	Bromelias	Agua Buena	94	94	15.4	15.4	0.002
Eug. championi	13	11	Bromelias	Bromelias	1	1	0	0	0.002
Eug. championi	13	11	Bromelias	Las Cruces	71.83	89.23	77.5	78.1	0.002
Eug. championi	13	11	La Gamba	Agua Buena	53.6	94.95	34.9	39.5	0.036
Eug. championi	13	11	La Gamba	Bromelias	66.65	94.54	50.4	53.2	0.002
Eug. championi	13	11	La Gamba	La Gamba	1	1	0	0	0.032
Eug. championi	13	11	La Gamba	Las Cruces	81.66	81.66	27.7	27.7	0.031
Eug. championi	13	11	La Gamba	Saladero	99.81	99.81	13.8	13.8	0.034
Eug. championi	13	11	Las Alturas	Agua Buena	69.53	72.57	80.7	81.9	0.067
Eug. championi	13	11	Las Alturas	Bromelias	74.75	74.75	95.5	95.5	0.002
Eug. championi	13	11	Las Alturas	La Gamba	69.5	69.5	48.5	48.5	0.032
Eug. championi	13	11	Las Alturas	Las Alturas	1	1	0	0	0.060
Eug. championi	13	11	Las Alturas	Las Cruces	62.09	62.09	22.5	22.5	0.056
Eug. championi	13	11	Las Alturas	Saladero	76.01	76.01	61	61	0.059
Eug. championi	13	11	Las Cruces	Agua Buena	62.05	88.2	62	64.4	0.064
Eug. championi	13	11	Las Cruces	Las Cruces	1	1	0	0	0.055
Eug. championi	13	11	Saladero	Agua Buena	23.06	92.42	21	33.9	0.067
Eug. championi	13	11	Saladero	Bromelias	53.96	92.89	36.1	47.6	0.002
Eug. championi	13	11	Saladero	Las Cruces	90.31	90.31	41.7	41.7	0.056
Eug. championi	13	11	Saladero	Saladero	1	1	0	0	0.061
Eug. flammea	14	8	La Gamba	La Gamba	1	1	0	0	0.167
Eug. flammea	14	8	La Gamba	Las Alturas	69.5	69.5	48.5	48.5	0.175
Eug. flammea	14	8	Las Alturas	La Gamba	69.5	69.5	48.5	48.5	0.163
Eug. flammea	14	8	Las Alturas	Las Alturas	1	1	0	0	0.163
Eug. flammea	14	8	Las Alturas	Las Cruces	62.09	62.09	22.5	22.5	0.054
Eug. flammea	14	8	Las Cruces	La Gamba	81.66	81.66	27.7	27.7	0.055
Eug. flammea	14	8	Las Cruces	Las Cruces	1	1	0	0	0.045
Eug. flammea	14	8	Las Cruces	Saladero	90.31	90.31	41.7	41.7	0.013
Eug. imperialis	15	20	Agua Buena	Agua Buena	1	1	0	0	0.064
Eug. imperialis	15	20	Bromelias	Agua Buena	94	94	15.4	15.4	0.057
Eug. imperialis	15	20	Bromelias	Bromelias	1	1	0	0	0.054
Eug. imperialis	15	20	La Gamba	Agua Buena	53.6	94.95	34.9	39.5	0.025
Eug. imperialis	15	20	La Gamba	Bromelias	66.65	94.54	50.4	53.2	0.020

MOLECULAR ECOLOGY

Eug. imperialis	15	20	La Gamba	La Gamba	1	1	0	0	0.020
Eug. imperialis	15	20	La Gamba	Saladero	99.81	99.81	13.8	13.8	0.016
Eug. imperialis	15	20	Saladero	Agua Buena	23.06	92.42	21	33.9	0.032
Eug. imperialis	15	20	Saladero	Bromelias	53.96	92.89	36.1	47.6	0.027
Eug. imperialis	15	20	Saladero	Saladero	1	1	0	0	0.028

Table S3. For each species, its body size, the number of orchid morphospecies visited, the site pairs between which genetic distances were calculated (Site 1 & Site 2), the percent of the distance between them that was forested when calculated using Euclidian paths (For. (E)), the percent of the distance between them that was forested when calculated using Broken-stick paths (For. (BS)), the Euclidian geographic distance between them (Km (E)), the Broken-stick geographic distance between them (Km (BS)), and the average genetic distance among individuals between those pairs (Hamming genetic distance).

MOLECULAR ECOLOGY

Species	Model	AIC	AICc	Δ AICc	Model Summary
Eug. sapphirina	**Full (E)**	**-39633.7**	**-39633.69**	**0**	Km (E) = 0.000058<br>For (E) = -0.00017<br><br>t = 7.82, P < 0.001<br>t = -49.5, P < 0.001
	Km (E)	-37599.99	-37599.99	-2033.7	
	For (E)	-39574.77	-39574.77	-58.92	
	Intercept	-37355.1	-37355.09	-2278.6	
	Full (BS)	-39365.78	-39365.77	-267.92	
	Km (BS)	-37584.11	-37584.1	-2049.59	
	For (BS)	-39077.91	-39077.9	-555.79	
Eug. dodsoni	Full (E)	-11777.71	-11777.68	-27.88	Km (BS) = -0.000067<br>For (BS) = -0.00012<br><br>t = -2.12, P = 0.036<br>t = -13.0, P < 0.001
	Km (E)	-11639.4	-11639.38	-166.18	
	For (E)	-11761.27	-11761.25	-44.31	
	Intercept	-11436.86	-11436.84	-368.72	
	**Full (BS)**	**-11805.59**	**-11805.56**	**0**	
	Km (BS)	-11645.41	-11645.39	-160.17	
	For (BS)	-11803.22	-11803.2	-2.36	
Eug. championi	Full (E)	-47630.4	-47595.95	-47630.4	Km (BS) = -0.000064<br>For (BS) = -0.00013<br><br>t = -11.1, P < 0.001<br>t = -31.3, P < 0.001
	Km (E)	-46906.2	-46878.64	-46906.2	
	For (E)	-47417.6	-47390.04	-47417.6	
	Intercept	-45447.31	-45426.64	-45447.31	
	**Full (BS)**	**-48031.57**	**-47997.12**	**-48031.56**	
	Km (BS)	-47116.26	-47088.7	-47116.25	
	TreeBS	-47912.54	-47884.98	-47912.53	
Eug. flammea	**Full (E)**	**-1438.61**	**-1438.406**	**0**	Km (E) = 0.00057<br>For (E) = -0.00085<br><br>t = 6.0, P < 0.001<br>t = -16.1, P < 0.001
	Km (E)	-1256.073	-1255.937	-182.469	
	For (E)	-1406.346	-1406.21	-32.196	
	Intercept	-1223.928	-1223.847	-214.559	
	**Full (BS)**	**-1438.61**	**-1438.406**	**0**	
	Km (BS)	-1256.073	-1255.937	-182.469	
	TreeBS	-1406.346	-1406.21	-32.196	

MOLECULAR ECOLOGY

Eug. imperialis	Full (E)	-26737.65	-26737.63	-338.3	Km (BS) = -0.00021<br>Km (BS) = -0.000049<br><br>*t* = -26.3, *P* < 0.001<br>*t* = -12.0, *P* < 0.001
	Km (E)	-26728.77	-26728.76	-347.17	
	For (E)	-25295.59	-25295.58	-1780.35	
	Intercept	-24822.72	-24822.71	-2253.22	
	**Full (BS)**	**-27075.95**	**-27075.93**	**0**	
	Km (BS)	-26936.14	-26936.13	-139.8	
	For (BS)	-26444.21	-26444.19	-631.74	

Table S4. Results from Maximum Likelihood of Population Effects (MLPE) models assessing the joint effects of the amount of land that was forested and geographic distance among site pairs on genetic distance among pairs of individuals. For each species, seven models were compared, a full model (Full (E)) that included as fixed effects the Euclidian geographic distance and amount of land that was forested along that path among site pairs, a model that included only Euclidian geographic distance (Geo (E)), a model that included only the amount of land that was forested (For (E)), a full model (Full (BS)) that included as fixed effects the Broken-stick geographic distance and amount of land that was forested along that path among site pairs, a model that included only Broken-stick geographic distance (Geo (BS)), a model that included only the amount of land that was forested (For (E)), and an intercept-only model (Intercept). Columns 3-6 show AIC and sample-size corrected AIC (AICc) values, the difference in AICc from the best model, and model results, including estimates for fixed effects and associated t and P-values.

MOLECULAR ECOLOGY

Species	Model	AIC	AICc	Δ AICc	Model Summary
Eug. sapphirina	**Full (E)**	**-27921.08**	**-27921.07**	**0**	Km (E) = 0.00013<br>For (E) = -0.000065<br><br>*t* = 11.9, *P* < 0.001<br>*t* = -9.82, *P* < 0.001
	Km (E)	-27827.86	-27827.85	-93.22	
	For (E)	-27785.01	-27785	-136.07	
	Intercept	-27645.89	-27645.89	-275.18	
	Full (BS)	-27826.33	-27826.32	-94.75	
	Km (BS)	-27828.12	-27828.11	-92.96	
	TreeBS	-27644.9	-27644.89	-276.18	
Eug. dodsoni	**Full (E)**	**-8385.668**	**-8385.625**	**0**	Km (E) = 0.00016<br>For (E) = 0.00011<br><br>*t* = 4.0, *P* < 0.001<br>*t* = 4.2, *P* < 0.001
	Km (E)	-8370.469	-8370.441	-15.184	
	For (E)	-8371.935	-8371.907	-13.718	
	Intercept	-8357.691	-8357.674	-27.951	
	Full (BS)	-8383.545	-8383.502	-2.123	
	Km (BS)	-8361.176	-8361.147	-24.478	
	TreeBS	-8385.064	-8385.036	-0.589	
Eug. championi	**Full (E)**	**-40852.16**	**-40852.15**	**0**	Km (E) = 0.000068<br>For (E) = -0.00012<br><br>*t* = 11.3, *P* < 0.001<br>*t* = -16.5, *P* < 0.001
	Km (E)	-40589.04	-40589.04	-263.11	
	For (E)	-40728.96	-40728.95	-123.2	
	Intercept	-40529.75	-40529.74	-322.41	
	Full (BS)	-40617.49	-40617.48	-234.67	
	Km (BS)	-40593.78	-40593.77	-258.38	
	TreeBS	-40588.87	-40588.86	-263.29	
Eug. flammea	**Full (E)**	**-1266.913**	**-1266.63**	**0**	Km (E) = 0.0032<br>For (E) = 0.04<br><br>*t* = 40.4, *P* < 0.001<br>*t* = -35.2, *P* < 0.001<br><br>*No paths went over water for this species, so Full (E) and Full (BS) are identical
	Km (E)	-1000.5586	-1000.3709	-266.2591	
	For (E)	-950.5323	-950.3445	-316.2855	
	Intercept	-949.1541	-949.0419	-317.5881	
	**Full (BS)***	**-1266.913**	**-1266.63**	**0**	
	Km (BS)	-1000.5586	-1000.3709	-266.2591	
	TreeBS	-950.5323	-950.3445	-316.2855	

MOLECULAR ECOLOGY

Eug. imperialis	**Full (E)**	**-20769.57**	**-20769.55**	**0**	Km (E) = 0.000075<br>For (E) = 0.00024<br><br>*t* = 4.4, *P* < 0.001<br>*t* = 28.1, *P* < 0.001
	Km (E)	-20074.27	-20074.26	-695.29	
	For (E)	-20752.27	-20752.26	-17.29	
	Intercept	-19647.13	-19647.12	-1122.43	
	Full (BS)	-20754.82	-20754.79	-14.76	
	Km (BS)	-20291.12	-20291.1	-478.45	
	For (BS)	-20455.06	-20455.04	-314.51	

Table S5. Results from Maximum Likelihood of Population Effects (MLPE) models assessing the joint effects of the amount of land that was forested and geographic distance among site pairs on genetic distance among pairs of individuals, using a dataset that included only genetic distances estimated from individuals at different sites. For each species, seven models were compared, a full model (Full (E)) that included as fixed effects the Euclidian geographic distance and amount of land that was forested along that path among site pairs, a model that included only Euclidian geographic distance (Geo (E)), a model that included only the amount of land that was forested (For (E)), a full model (Full (BS)) that included as fixed effects the Broken-stick geographic distance and amount of land that was forested along that path among site pairs, a model that included only Broken-stick geographic distance (Geo (BS)), a model that included only the amount of land that was forested (For (E)), and an intercept-only model (Intercept). Columns 3-6 show AIC and sample-size corrected AIC (AICc) values, the difference in AICc from the best model, and model results, including estimates for fixed effects and associated t and P-values.

MOLECULAR ECOLOGY

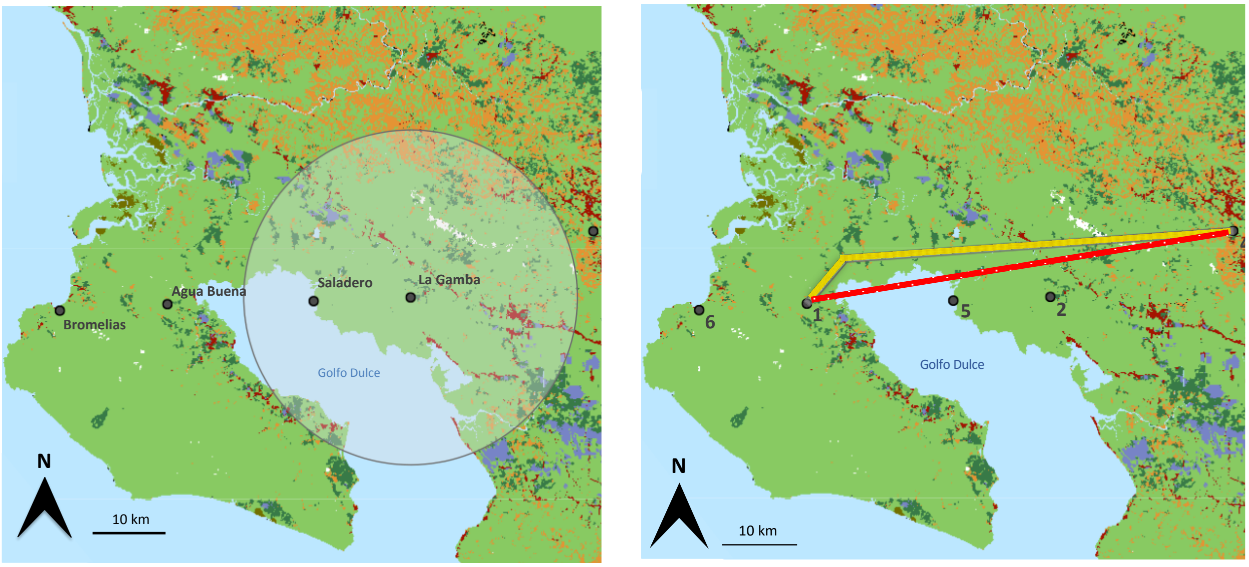

Figure S1. *Left panel:* The percent forest within a circle of radius 24 km was calculated using GIS. Light green = pastureland; dark green = forest; blue = water. *Right panel:* The percent forest between pairs of sites was calculated using both Euclidian (yellow) and Broken-stick (red) paths.
